# Supplementary material for: Role and mechanism of NCAPD3 in promoting malignant behaviors in gastric cancer
Source: Front Pharmacol. 2024 Apr 22;15:1341039. doi: 10.3389/fphar.2024.1341039 (PMC11070777; doi:10.3389/fphar.2024.1341039)
Supplement: Supplementary file 11 [file DataSheet2.ZIP › GSEA/Transcipt factor/my_analysis.Gsea.1599461814734/AREB6_03.html]

Details for gene set AREB6\_03[GSEA]

|  || Dataset | filtered\_dataset.sample\_info.cls#WT\_versus\_NCAPD3\_MUT |
| Phenotype | sample\_info.cls#WT\_versus\_NCAPD3\_MUT |
| Upregulated in class | NCAPD3\_MUT |
| GeneSet | AREB6\_03 |
| Enrichment Score (ES) | -0.43413037 |
| Normalized Enrichment Score (NES) | -1.999295 |
| Nominal p-value | 0.002386635 |
| FDR q-value | 0.06646059 |
| FWER p-Value | 0.586 |
Table: GSEA Results Summary

  

Fig 1: Enrichment plot: AREB6\_03      
 Profile of the Running ES Score & Positions of GeneSet Members on the Rank Ordered List

  

| SYMBOL | TITLE | RANK IN GENE LIST | RANK METRIC SCORE | RUNNING ES | CORE ENRICHMENT || 1 | 55132 | LARP1B | 189 | 0.686 | -0.0661 | No |
| 2 | 79651 | RHBDF2 | 422 | 0.519 | -0.1802 | No |
| 3 | 23235 | SIK2 | 523 | 0.460 | -0.2054 | No |
| 4 | 11091 | WDR5 | 822 | 0.304 | -0.3888 | No |
| 5 | 667 | DST | 886 | -0.303 | -0.4033 | Yes |
| 6 | 55652 | SLC48A1 | 897 | -0.317 | -0.3782 | Yes |
| 7 | 3664 | IRF6 | 908 | -0.334 | -0.3514 | Yes |
| 8 | 3691 | ITGB4 | 926 | -0.352 | -0.3278 | Yes |
| 9 | 2317 | FLNB | 936 | -0.360 | -0.2976 | Yes |
| 10 | 79094 | CHAC1 | 949 | -0.367 | -0.2689 | Yes |
| 11 | 7067 | THRA | 976 | -0.392 | -0.2477 | Yes |
| 12 | 10938 | EHD1 | 1019 | -0.419 | -0.2353 | Yes |
| 13 | 1657 | DMXL1 | 1080 | -0.462 | -0.2314 | Yes |
| 14 | 3918 | LAMC2 | 1088 | -0.466 | -0.1890 | Yes |
| 15 | 54852 | PAQR5 | 1151 | -0.515 | -0.1812 | Yes |
| 16 | 117178 | SSX2IP | 1163 | -0.532 | -0.1350 | Yes |
| 17 | 6310 | ATXN1 | 1177 | -0.547 | -0.0886 | Yes |
| 18 | 5154 | PDGFA | 1191 | -0.566 | -0.0404 | Yes |
| 19 | 23657 | SLC7A11 | 1205 | -0.581 | 0.0094 | Yes |
| 20 | 1788 | DNMT3A | 1253 | -0.641 | 0.0408 | Yes |
| 21 | 64854 | USP46 | 1300 | -0.702 | 0.0791 | Yes |
Table: GSEA details [plain text format]

  

Fig 2: AREB6\_03      
 Blue-Pink O' Gram in the Space of the Analyzed GeneSet

  

Fig 3: AREB6\_03: Random ES distribution      
 Gene set null distribution of ES for **AREB6\_03**
